# Prevalence and Predictors of Musculoskeletal Pain Among Pregnant Women: A Cross-Sectional Study

**DOI:** 10.3390/healthcare13192524

**Published:** 2025-10-05

**Authors:** Jalal Uddin, Shahida Sultana Shumi, Jason D. Flatt

**Affiliations:** 1 Department of Epidemiology and Biostatistics, School of Public Health, University of Nevada, Las Vegas, NV 89154, USA; 2 Department of Physiotherapy, Saic College of Medical Science and Technology, Dhaka 1216, Bangladesh; 3 Department of Social and Behavioral Health, School of Public Health, University of Nevada, Las Vegas, NV 89154, USA; jason.flatt@unlv.edu

**Keywords:** musculoskeletal pain, pregnancy, antenatal care, pain severity, trimester, BMI, ergonomic intervention

## Abstract

**Background**: Musculoskeletal (MSK) pain is a frequent but under-addressed concern during pregnancy. In Bangladesh, challenges such as limited antenatal care (ANC) access and heavy maternal workloads make this issue particularly urgent for maternal health. This study aimed to determine the prevalence and predictors of MSK pain among pregnant women attending government ANC clinics in Bangladesh. **Methods**: A facility-based cross-sectional study was conducted among 300 pregnant women recruited from two government hospitals in Dhaka Division. Data were collected using structured interviewer-administered questionnaires covering patient characteristics, pain-related characteristics, and pregnancy-related characteristics. Pain was measured using the Numeric Pain Rating Scale (NPRS; mild <4, moderate 4–7, severe >7), and body mass index (BMI) was calculated based on self-reported height and weight. Descriptive statistics, chi-square tests, and multivariable logistic regression were employed to identify factors independently associated with MSK pain. **Results**: Overall, 67% of women reported MSK pain, most frequently in the lower back and lower abdomen. Women in later trimesters had about twice the odds of experiencing pain, while those with obesity had nearly six times higher odds compared to women with normal body mass index (BMI). **Conclusions**: MSK pain is common among pregnant women in Bangladesh and shows associations with later gestational stages and obesity. These findings suggest that integrating routine screening and non-pharmacological management into ANC may help support maternal health and reduce preventable complications in resource-limited settings.

## 1. Introduction

Musculoskeletal (MSK) pain is a common health issue during pregnancy, affecting up to 70% of expectant mothers globally, with lower back pain being the most frequently reported symptom [1]. This pain ranges from mild to severe, disabling conditions that disrupt daily functioning, mobility, and overall quality of life [2]. MSK pain has been associated with increased maternal stress, sleep disturbances, and diminished well-being during pregnancy [3]. If left unaddressed, it may continue into the postpartum period and be associated with complications such as pelvic floor dysfunction, functional limitations, and depressive symptoms, which can contribute to slower maternal recovery [4,5,6,7].

Several physiological and biomechanical factors contribute to the development of MSK pain in pregnancy. Rising levels of relaxin, a hormone essential for childbirth preparation, can lead to joint laxity and increased musculoskeletal strain [8]. These changes, along with gestational weight gain and altered posture, shift the body’s center of gravity and place added stress on the lumbar spine and pelvic region [9]. Maternal characteristics such as age and body mass index (BMI) are relevant; for example, up to 90% of women with severe obesity (BMI ≥ 40) report musculoskeletal pain, with the knee (63.5%) and lumbar spine (46.8%) most affected, and pain severity increasing proportionally with BMI [10,11]. Socioeconomic and environmental contexts also influence the burden of MSK pain. Studies have shown that women with lower education levels and those engaged in manual occupations experience a higher prevalence of musculoskeletal problems during pregnancy, with limited access to healthcare further compounding the risk [12,13]. Recent evidence from rural Nigeria also highlights that labor-intensive domestic and unpaid work is strongly associated with musculoskeletal disorders among women, emphasizing the role of occupational and employment-related factors [14].

In Bangladesh, these challenges are shaped by both health system gaps and women’s everyday realities. While ANC coverage has gradually improved, only about one-third of women complete the recommended four visits, often due to issues such as limited facility readiness, staff shortages, and variable service quality [15]. For women in rural or disaster-prone areas, the distance to health facilities and lower availability of institutional care add further barriers [16]. Daily life also plays a role; many women continue with demanding domestic and agricultural work throughout pregnancy, usually without guidance on safe posture or rest [16]. At the same time, rehabilitation and physiotherapy remain under-recognized within routine maternal healthcare, leaving women with few accessible options for safe, non-pharmacological support [17].

Although MSK pain in pregnancy has been extensively studied in high-income countries, limited evidence exists for low-resource environments such as Bangladesh. Cultural norms, healthcare accessibility, and physical workload may uniquely impact the prevalence and experience of MSK pain in this context [12]. Given the limited evidence in low-resource settings, this study aimed to estimate the prevalence and predictors of pregnancy-related MSK pain among ANC attendees in Bangladesh. Unlike prior global research, this study specifically examines how local socioeconomic, occupational, and anthropometric factors, such as obesity, may influence MSK pain risk. The findings may inform context-specific approaches to integrate MSK screening, ergonomic counseling, and physiotherapy referral into routine ANC services.

## 2. Methods

### 2.1. Study Design and Setting

This cross-sectional study was conducted from April to September 2019 at two government-run healthcare institutions in Bangladesh: Dhaka Medical College Hospital (DMCH), the country’s largest tertiary referral center, and the 100-Bed Zilla Hospital in Narsingdi, a district-level hospital primarily serving rural and semi-urban populations. These facilities were selected for their high patient volumes and ability to represent diverse maternal demographics. The study received ethical clearance from the Institutional Review Board of SAIC College of Medical Science and Technology (protocol Phy/SIMT/17/04/2019; approval date 17 April 2019), and written administrative approvals were obtained from each participating institution.

### 2.2. Study Population

The study recruited pregnant women attending antenatal outpatient departments at the two hospitals during routine clinical visits. Participants were consecutively invited to join the study regardless of gestational age, ensuring natural variation across trimesters. Inclusion criteria were age ≥ 18 years, confirmed pregnancy, and provision of informed written consent. Women with pre-existing musculoskeletal disorders unrelated to pregnancy (e.g., arthritis, chronic back injuries) were screened through clinical history and excluded to minimize confounding. A total of 300 participants were enrolled using convenience sampling. This approach was adopted because Bangladesh lacks a centralized ANC registry or sampling frame that would allow random selection of pregnant women. Convenience sampling enabled timely recruitment across both a tertiary and a district hospital, capturing women from diverse urban, rural, and semi-urban settings. While this limits representativeness, it provided a pragmatic strategy for generating initial evidence on pregnancy-related MSK pain in Bangladesh.

### 2.3. Data Collection

Data were collected through structured, face-to-face interviews conducted in Bangla by trained female research assistants. Interviews were held in private consultation rooms to ensure confidentiality and comfort. All interviewers received standardized training on the use of the data collection tool to ensure consistent administration. The questionnaire was pilot tested on a subsample (*n* = 15) to ensure clarity, cultural appropriateness, and feasibility of administration. Due to the small pilot sample size, no formal reliability index (e.g., Cronbach’s alpha) was calculated. Although this limited our ability to quantify internal consistency, the questionnaire was adapted from previously validated instruments and refined during pilot testing to enhance cultural relevance and comprehensibility, as described in Section 2.4. Data collection spanned six months, providing a representative snapshot of antenatal clinic attendees across both sites.

### 2.4. The Study Instrument

The structured questionnaire consisted of three main sections: patient characteristics, pain-related characteristics, and pregnancy-related characteristics. The patient characteristics section included variables such as age, place of residence, educational attainment, family type, household income, occupation, and BMI, chosen for their relevance to maternal health and MSK research [10,11,12]. BMI was calculated from self-reported height and weight and categorized as: underweight (<18.5), normal (18.5–24.9), overweight (25.0–29.9), and obese (≥30.0) [11,12]. Pain-related variables included the presence, duration, nature and severity of pain (e.g., sharp, dull, burning), pain location, pain referral, and pattern [1,18]. Lower abdominal pain was retained in the case definition by design to reflect programmatic ANC screening practices, although we acknowledge in the Limitations that this may inflate prevalence estimates. Pain severity was assessed using the Numeric Pain Rating Scale (NPRS), a validated self-report tool that allows individuals to rate their pain on a scale from 0 (no pain) to 10 (worst possible pain) [19]. Pain severity was further categorized into mild (<4), moderate (4–7), and severe (>7) levels [20,21]. Pregnancy-specific factors, such as the current trimester, pain during urination, and previous pregnancy history, were documented due to their association with varying degrees of MSK pain during pregnancy [1,4].

### 2.5. Data Analysis

Data were analyzed using SPSS version 29. Descriptive statistics were calculated to summarize the patient characteristics, pain-related characteristics, and pregnancy-related characteristics. To assess MSK pain, participants reported pain across specific anatomical sites, including the neck, arm, forearm, hand, lower back, lower abdomen, leg, ankle, and foot. Responses were consolidated to determine overall MSK pain prevalence. Participants reporting pain in any of these sites were classified as experiencing MSK pain. This approach ensured that individuals with multiple pain sites were accurately categorized under a single MSK pain prevalence measure for analysis. Bivariate methods were used to examine associations between MSK pain and various patient characteristics. Chi-square tests were employed to analyze categorical variables, and independent *t*-tests were used to compare continuous variables, such as age. Multivariable logistic regression was conducted to identify significant predictors of MSK pain. Variables with a *p*-value < 0.20 in bivariate analyses were considered for entry into the multivariable logistic regression. In addition, variables with strong theoretical or clinical relevance (age, BMI, trimester, educational attainment, and history of miscarriage) were retained regardless of bivariate significance, consistent with established recommendations for predictor selection in logistic regression [22]. Multicollinearity was assessed using variance inflation factors (VIFs); all values were close to 1, confirming no collinearity concerns. All available data were analyzed. Sociodemographic and pregnancy-related variables had no missing observations. Among 300 participants, 216 reported musculoskeletal pain and therefore completed follow-up items on pain site, severity, and pattern. The remaining 84 without pain were not asked these questions, so the reduced denominators in SPSS output reflect conditional skip patterns rather than true missing data. No imputation was performed. Because participants were recruited from two hospitals (a tertiary referral center and a district hospital), we compared baseline characteristics and pain prevalence across sites. No major differences were observed, and therefore results are presented in pooled form. Adjusted odds ratios (aOR) and 95% confidence intervals (CI) are reported. A *p*-value of less than 0.05 was considered statistically significant. Model performance was evaluated using multiple fit indices. The Hosmer–Lemeshow goodness-of-fit test suggested adequate calibration (χ^2^ = 4.255, df = 8, *p* = 0.833). The −2 log likelihood was 357.114, and the model explained between 13.1% (Cox & Snell R^2^) and 17.9% (Nagelkerke R^2^) of the variance in MSK pain. Together, these results indicate that the final model demonstrated acceptable fit and explanatory power.

## 3. Results

### 3.1. Participant Characteristics

The mean age of participants was 24.3 years, with most younger than 25 years. A majority resided in rural areas, lived in extended families, and were housewives. Over half reported household income < 10,000 BDT, and nearly half were overweight or obese (Table 1).

### 3.2. Pain-Related Characteristics

Two-thirds of participants reported musculoskeletal pain. Most of them experienced pain for less than three months (64.0%), and the predominant pain areas were lower back pain (38.3%) and lower abdominal pain (36.3%). Pain was typically dull or moderate in severity and intermittent in pattern (Table 2).

### 3.3. Pregnancy-Related Characteristics

Nearly half of participants were in the second trimester, and a quarter reported a history of miscarriage. More than two-fifths had no previous pregnancies, and among those with childbirth history, cesarean section accounted for about one-quarter of deliveries (Table 3).

### 3.4. Bivariate Analysis

Musculoskeletal pain was significantly associated with several sociodemographic and pregnancy-related variables. Age was significantly higher among those with pain (mean 24.98 ± 4.65 years) compared to those without (mean 23.14 ± 4.44 years; *p* < 0.001). Rural residence (*p* = 0.04), extended family (*p* = 0.02), higher BMI (*p* < 0.001), being in the second or third trimester (*p* = 0.02), and a history of previous pregnancies (*p* = 0.04) were significantly associated with musculoskeletal pain (Table 4).

### 3.5. Multivariable Logistic Regression

Table 5 summarizes the results of the multivariable logistic regression model examining predictors of MSK pain among pregnant women.

After adjustment, obesity and trimester of pregnancy were the strongest predictors of MSK pain. Obese women had nearly sixfold higher odds compared with those of normal BMI (aOR = 5.73, 95% CI: 1.85–17.79, *p* = 0.003). Women in the second (aOR = 1.99, 95% CI: 1.07–3.70) and third trimesters (aOR = 2.18, 95% CI: 1.05–4.52) were about twice as likely to report pain compared with those in the first trimester.

Other sociodemographic and obstetric variables, including age, place of residence, educational attainment, family type, household income, previous history of pregnancy, history of miscarriage, and history of abortion, were not significantly associated with MSK pain.

## 4. Discussion

This study investigated the prevalence and correlates of MSK pain among pregnant women in Bangladesh, a lower-middle-income country (LMIC) [23]. These findings highlight the importance of considering non-invasive and non-pharmacological strategies as an integral part of antenatal care, since the use of medications during pregnancy is often constrained by safety concerns for both the mother and fetus [24].

In our study, approximately 67% of participants reported experiencing MSK pain, with most describing their symptoms as moderate (39.3%) or mild (24.7%). This prevalence is consistent with global estimates, which place pregnancy-related MSK pain between 45% and 75%, depending on the population and trimester studied [25,26]. The high prevalence of lower back pain (38.3%) and lower abdominal pain (36.3%) is also consistent with prior investigations [27]. These findings reinforce the need for early screening and interventions focusing on pain management and preventive strategies. Furthermore, contributing factors such as poor ergonomic posture, unadjusted work routines during pregnancy, and lack of awareness of self-care measures may intensify these discomforts if not addressed early on [28]. These regions are particularly vulnerable to biomechanical stress from increasing uterine weight, anterior pelvic tilt, and altered gait mechanics, all of which are associated with MSK pain during pregnancy [9].

Among individual predictors, age was initially associated with MSK pain but did not remain significant in the adjusted model, suggesting confounding by other demographic or clinical factors. Previous research has produced mixed findings, with some studies linking older age to greater pain due to reduced musculoskeletal resilience [29], while others reported negligible differences after adjusting for parity and physical activity [30]. In Bangladesh, limited prenatal resources may exacerbate age-related vulnerability, as older women could face compounded effects of physiological changes and inadequate support. Cross-cultural inconsistencies in the literature may also reflect variations in antenatal physical activity practices and access to pain management education [31].

In contrast, our finding that MSK pain increases in later trimesters is consistent with longitudinal studies [1,4] and likely reflects physiological changes such as relaxin-mediated joint laxity, increased lumbar lordosis, and redistribution of body weight that cumulatively strain the musculoskeletal system [32]. Dysuria, noted in some participants, may reflect urinary tract infection, which can also present with lower back pain [33]. This overlap could have modestly influenced reported MSK symptoms and should be considered in ANC assessment [34]. Interventional trials further suggest that initiating appropriate non-pharmacological strategies during the second trimester may help reduce pain severity in the third trimester [35,36].

BMI was also significantly associated with MSK pain, with obese participants reporting more frequent and severe symptoms. Biomechanically, excess body weight increases the axial load on the lumbar spine and lower extremities, exacerbating postural imbalances and is associated with greater fatigue and pain [10]. This finding appears consistent with prior studies highlighting the compounded effects of obesity and pregnancy on MSK morbidity [37]. Evidence suggests that multi-component, non-pharmacological interventions may help reduce both pain severity and adverse pregnancy outcomes in high-BMI groups [38].

In contrast to physiological predictors, place of residence, educational attainment, household income, and family type were not significantly associated with MSK pain in adjusted models. This suggests that physiological factors may play a more prominent role, though environmental influences should not be overlooked. For instance, women in rural areas often engage in physically demanding labor during pregnancy and may have reduced access to structured antenatal care (ANC) services [12,39]. The absence of statistical significance may also reflect underpowered subgroup analyses or the influence of unmeasured confounders such as occupation-specific physical load, domestic workload, or health literacy [40,41]. Similarly, variables related to Previous history of pregnancy, miscarriage, abortion, and childbirth were not significant in adjusted models, even though some showed associations in bivariate analyses. This indicates that reproductive history may not independently predict MSK pain once other risk factors are accounted for.

Globally, non-pharmacological interventions, including physiotherapy, structured exercise, ergonomic counseling, and antenatal sessions, have been shown to reduce lumbar and pelvic pain, improve function, and support adherence through social support [42]. Such non-pharmacological strategies are particularly important in pregnancy, where medication use is restricted, and they remain feasible and adaptable in low-resource settings.

Our findings, therefore, highlight important considerations for public health practice. Routine MSK pain screening could be explored within ANC, particularly during the second and third trimesters when symptoms most commonly emerge. Incorporating validated tools such as the Numeric Pain Rating Scale (NPRS) and site-specific pain maps may help improve diagnostic precision. Non-pharmacological strategies—including physiotherapy, exercise-based programs, yoga, posture correction, and self-management techniques—could be feasibly piloted within ANC to support maternal comfort and quality of life. Evidence from Indonesia supports the acceptability of such programs in low-income contexts [43]. In addition, training healthcare providers to identify and manage MSK pain in culturally sensitive ways may enhance the quality of maternal care [3]. Primary care providers and midwives could also be equipped to recognize MSK symptoms early and initiate timely referrals to physiotherapy or lifestyle-based interventions to prevent progression of chronic pain.

At the systems level, these results suggest opportunities for incorporating MSK pain management into national ANC guidelines to promote equity and sustainability. In Bangladesh, however, implementation faces barriers such as limited ANC access in rural areas, shortages of trained rehabilitation providers, and cultural norms that expect women to continue heavy domestic or agricultural workloads during pregnancy [44]. Addressing these challenges is essential for the eventual integration of MSK care into national programs. Evidence from systematic reviews suggests that early implementation of physiotherapy interventions, including targeted exercise and ergonomic guidance, may improve outcomes for pregnancy-related MSK conditions [45]. Additionally, international expert consensus highlights postural education, strengthening exercises, and broader lifestyle considerations as essential elements in managing MSK pain, which could be delivered through scalable platforms such as mobile health [42,46].

## 5. Limitations

This study has several limitations. The use of convenience sampling from two public hospitals may limit generalizability, particularly to private-sector or remote rural settings. This pragmatic approach was necessary given the absence of a national ANC registry or sampling frame in Bangladesh, but we acknowledge that it reduces representativeness. BMI was calculated from self-reported height and weight, which are subject to recall and misclassification bias. This approach was adopted due to the very high patient load and limited resources in the study hospitals, where direct measurement was not feasible. Gestational weight gain was not systematically recorded in hospital registers and therefore could not be analyzed as a predictor; this is noted as an important area for future research. The questionnaire was pilot tested for clarity and feasibility, but no formal reliability index (e.g., Cronbach’s alpha) was calculated, which may affect internal consistency. Although the multivariable models adjusted for several potential confounders, residual confounding from unmeasured factors such as pre-pregnancy physical activity, occupation, or psychosocial stressors cannot be ruled out. Additionally, postural defects or other musculoskeletal risk factors were not systematically verified, which may have introduced residual confounding. For site-specific pain and severity variables, denominators were lower because only women reporting musculoskeletal pain (*n* = 216) completed these follow-up items; this reflects the survey’s skip pattern rather than true missing data, though it modestly reduced precision for these analyses. The broad definition of MSK pain, which included abdominal symptoms, may also have inflated prevalence estimates compared with studies limited to lumbopelvic pain. Another limitation is the time gap between data collection (2019) and publication, as the COVID-19 pandemic disrupted the subsequent steps of data entry, cleaning, analysis, and manuscript preparation, leading to delays in submission. Although no major differences were observed between the tertiary and district hospitals, unmeasured site-level factors (e.g., variations in patient flow or local practice context) could still have influenced responses. Finally, the cross-sectional design precludes causal inference. Future research should therefore adopt longitudinal designs to monitor the trajectory of MSK pain across pregnancy and postpartum, and rigorously evaluate the effectiveness of tailored non-pharmacological interventions such as structured exercise, ergonomic education, and digital health tools in South Asian contexts.

## 6. Conclusions

MSK pain is a highly prevalent and clinically significant issue among pregnant women in Bangladesh. Trimester of pregnancy, BMI, and to a lesser extent, age were associated with occurrence, while variables such as place of residence, educational attainment, household income, family type, and previous pregnancy history did not show independent associations in adjusted analyses. Given these findings, routine MSK screening and non-pharmacological interventions should be incorporated into ANC protocols to enhance maternal comfort, mobility, and quality of life. Beyond individual-level interventions, this study underscores the need to strengthen antenatal care systems, ensure equitable access to screening and management, and raise awareness of pregnancy-related MSK pain. Practical steps could include training midwives to deliver ergonomic counseling, integrating physiotherapy into referral pathways, and leveraging mobile health platforms for screening and follow-up. For example, a 5 min midwife-delivered posture script and a single-page exercise handout provided at the 2nd ANC visit could serve as low-cost, scalable strategies. Addressing these challenges through safe, non-invasive strategies should be recognized as a public health priority. Policymakers, clinicians, and researchers should act collectively to integrate evidence-based, non-pharmacological approaches into maternal health programs to improve comfort, mobility, and long-term outcomes for mothers.

## Figures and Tables

**Table 1:** Participant Characteristics.

Category	Subcategory	Count (*n*)	Percentage (%)
**Sociodemographic characteristics**			
**Age**	<20	93	31
	20–25	94	31.3
	25–30	83	27.7
	>30	30	10
**Mean ± SD (range)**	24.27 ± 4.65 years (18–35)		
**Place of residence**	Urban	114	38
	Rural	181	60.3
	Semi-Urban	5	1.7
**Educational attainment**	Illiterate	3	1
	Can sign only	14	4.7
	Primary Education	106	35.3
	Secondary Education	80	26.7
	Higher Secondary Education	46	15.3
	College Degree	41	13.7
	Other Education	10	3.3
**Family Type**	Extended Family	167	55.7
	Nuclear Family	133	44.3
**Economic characteristics**			
**Monthly household Income**	<10,000 BDT	155	51.7
	10,000–20,000 BDT	101	33.7
	20,000–30,000 BDT	31	10.3
	>30,000 BDT	13	4.3
**Occupation**	Housewife	265	88.3
	Student	13	4.3
	Government Service	4	1.3
	Teacher	2	0.7
	Other Occupation	16	5.3
**Anthropometric characteristics**			
**BMI**	Underweight (<18.5)	15	5
	Normal (18.5–25)	138	46
	Overweight (25–29.9)	106	35.3
	Obese (≥30.0)	41	13.7

**Table 2:** Pain-related Characteristics.

Category	Subcategory	Count (*n*)	Percentage (%)
**Pain**	Yes	201	67
**Pain duration**	<3 months	192	64
	>3 months	24	8
**Nature of pain**	Sharp	83	27.7
	Dull	107	35.7
	Burning	5	1.7
	Shooting	17	5.7
	Others	4	1.3
**Severity of pain**	Mild < 4	74	24.7
	Moderate (4–7)	118	39.3
	Severe > 7	24	8
**Pain area**	Referred pain	71	23.7
	Headache	30	10
	Neck pain	5	1.7
	Arm pain	8	2.7
	Forearm pain	8	2.7
	Hand pain	7	2.3
	Lower back pain	115	38.3
	Lower abdominal pain	109	36.3
	Leg pain	55	18.3
	Ankle pain	16	5.3
	Foot pain	13	4.3
**Pain pattern**	Constant	30	10
	Intermittent	186	62

Note: ‘Pain area’ was a multiple-response item; therefore, totals may exceed N = 201 and percentages may not sum to 100%.

**Table 3:** Pregnancy-related Characteristics.

Category	Subcategory	Count (*n*)	Percentage (%)
**Trimester**	1st Trimester	76	25.3
	2nd Trimester	143	47.7
	3rd Trimester	81	27
**Pain during urination**	Yes	53	17.7
	No	247	82.3
**Previous history of pregnancy**	Single pregnancy	106	35.3
	Multiple pregnancies	70	23.3
	No previous history	124	41.3
**Previous history of miscarriage**	Yes	78	26
	No	222	74
**History of abortion**	Yes	20	6.7
	No	280	93.3
**Previous history of childbirth**	Normal vaginal delivery	95	31.7
	Assisted delivery	3	1
	Cesarean section	77	25.7

**Table 4:** Bivariate Analysis of Patient Characteristics and Musculoskeletal Pain.

Variables	Musculoskeletal Pain *n* (%) N = 201 (67.0)	No Musculoskeletal Pain *n* (%) N = 99 (33.0)	*p*-Value
Age, Mean (SD)	24.98 (4.65)	23.14 (4.44)	**<0.001**
**Area**			**0.04**
Urban	82 (44.3%)	37 (32.2%)	
Rural	103 (55.7%)	78 (67.8%)	
**Education**			0.42
Under College Degree	122 (65.9%)	81 (70.4%)	
College Degree	63 (34.1%)	34 (29.6%)	
**Income**			0.08
<25,000 BDT	156 (84.3%)	105 (91.3%)	
>25,000 BDT	29 (15.7%)	10 (8.7%)	
**Family Types**			**0.02**
Nuclear	92 (49.7%)	41 (35.7%)	
Extended	93 (50.3%)	74 (64.3%)	
**Trimester of pregnancy**			**0.02**
First trimester	37 (20.0%)	39 (33.9%)	
Second trimester	92 (49.7%)	51 (44.3%)	
Third trimester	56 (30.3%)	25 (21.7%)	
**Previous history of pregnancy**			**0.04**
Single	71 (38.4%)	35 (30.4%)	
Multiple	48 (25.9%)	22 (19.1%)	
None of them	66 (35.7%)	58 (50.4%)	
**Previous history of miscarriage**			0.81
Yes	49 (26.5%)	29 (25.2%)	
No	136 (73.5%)	86 (74.8%)	
**Previous History of Abortion**			0.20
Yes	15 (8.1%)	5 (4.3%)	
No	170 (91.9%)	110 (95.7%)	
**BMI Category**			**<0.001**
Underweight	8 (4.3%)	7 (6.1%)	
Normal	76 (41.1%)	62 (53.9%)	
Overweight	64 (34.6%)	42 (36.5%)	
Obese	37 (20.0%)	4 (3.5%)	

Note: Values are mean (SD) or *n* (%). *p*-values from *t*-tests (continuous) and chi-square tests (categorical).

**Table 5:** Logistic Regression Analysis of Factors Associated with Musculoskeletal Pain.

Variables	aOR	95% CI (Lower–Upper)	*p*-Value
**Age**	1.072	0.992–1.158	0.08
**Place of residence** (Ref: Urban)	0.824	0.477–1.425	0.49
**Educational attainment** (Ref: ≤College)	0.903	0.495–1.645	0.74
**Family type** (ref: nuclear)	0.620	0.358–1.077	0.09
**Household monthly income** (>25,000 BDT)	1.789	0.776–4.126	0.17
**BMI category** (ref: normal)			**0.02**
– Underweight	0.937	0.302–2.909	0.91
– Overweight	0.994	0.565–1.748	0.98
– Obese	5.733	1.847–17.792	**0.003**
**Trimester of pregnancy** (Ref: 1st Trimester)			
– 2nd Trimester	1.990	1.071–3.698	**0.03**
– 3rd Trimester	2.176	1.047–4.521	**0.04**
**Previous history of pregnancy** (ref: single)			
– Multiple	0.624	0.283–1.374	0.24
– No history of pregnancy	0.742	0.397–1.384	0.35
**Previous history of miscarriage** (Yes vs. No)	0.644	0.341–1.215	0.17
**Previous history of abortion** (Yes vs. No)	1.467	0.460–4.674	0.52

Notes: aOR = Adjusted Odds Ratio from multivariable logistic regression. Reference categories are specified for categorical variables. *p*-values indicate statistical significance (*p* < 0.05).

## Data Availability

The data presented in this study are available on request from the corresponding author. The data are not publicly available due to ethical restrictions and protection of participant confidentiality.

## References

[1] Salari N., Mohammadi A., Hemmati M., Hasheminezhad R., Kani S., Shohaimi S., Mohammadi M. (2023). The global prevalence of low back pain in pregnancy: A comprehensive systematic review and meta-analysis. BMC Pregnancy Childbirth.

[2] Smith M.W., Marcus P.S., Wurtz L.D. (2008). Orthopedic issues in pregnancy. Obstet. Gynecol. Surv..

[3] Kesikburun S., Güzelküçük Ü., Fidan U., Demir Y., Ergün A., Tan A.K. (2018). Musculoskeletal pain and symptoms in pregnancy: A descriptive study. Ther. Adv. Musculoskelet. Dis..

[4] Thabah M., Ravindran V. (2015). Musculoskeletal problems in pregnancy. Rheumatol. Int..

[5] Sakamoto A., Gamada K. (2019). Altered musculoskeletal mechanics as risk factors for postpartum pelvic girdle pain: A literature review. J. Phys. Ther. Sci..

[6] Schaffir J., Kunkler A., Lynch C.D., Benedict J., Soma L., Doering A. (2018). Association between postpartum physical symptoms and mood. J. Psychosom. Res..

[7] Li M., Li D., Bu J., Zhang X., Liu Y., Wang H., Wu L., Song K., Liu T. (2024). Examining the factors influencing postpartum musculoskeletal pain: A thorough analysis of risk factors and pain assessment indices. Eur. Spine J..

[8] Bernstein C., Takoudes T.C. (2017). Medical Problems During Pregnancy: A Comprehensive Clinical Guide.

[9] Conder R., Zamani R., Akrami M. (2019). The biomechanics of pregnancy: A systematic review. J. Funct. Morphol. Kinesiol..

[10] MacLellan G.A., Dunlevy C., O’Malley E., Blake C., Breen C., Gaynor K., Wallace N., Yoder R., Casey D., Mehegan J. (2017). Musculoskeletal pain profile of obese individuals attending a multidisciplinary weight management service. Pain.

[11] Rosa S., Martins D., Martins M., Guimarães B., Cabral L., Horta L. (2021). Body mass index and musculoskeletal pain: A cross-sectional study. Cureus.

[12] Ramachandra P., Maiya A.G., Kumar P., Kamath A. (2015). Prevalence of musculoskeletal dysfunctions among Indian pregnant women. J. Pregnancy.

[13] Yasobant S., Nibedita S., Saswata S., Arnansu M., Kirti S. (2014). Musculoskeletal problems among pregnant women: A facility-based survey in Odisha. Int. J. Med. Res. Health Sci..

[14] Osinuga A., Fethke N.B., Story W.T., Ibitoye S.E., Baker K.K. (2022). Assessing the relationship between domestic work experience and musculoskeletal health among rural Nigerian women. PLoS ONE.

[15] Khan M.N., Alam M.B., Chowdhury A.R., Kabir M.A., Khan M.M.A. (2024). Availability and readiness of healthcare facilities and their effects on antenatal care services uptake in Bangladesh. BMC Health Serv. Res..

[16] Begum A., Hamid S.A. (2023). Maternal healthcare utilization in rural Bangladesh: A comparative analysis between high and low disaster-prone areas. PLoS Glob. Public Health.

[17] Mamin F.A., Hayes R. (2018). Physiotherapy in Bangladesh: Inequality begets inequality. Front. Public Health.

[18] Stretanski M.F., Stinocher S., Grandhe S. (2025). Pain Assessment. StatPearls [Internet].

[19] Hawker G.A., Mian S., Kendzerska T., French M. (2011). Measures of adult pain: Visual Analog Scale, Numeric Rating Scale, McGill Pain Questionnaire, Short-Form McGill Pain Questionnaire, Chronic Pain Grade Scale, SF-36 Bodily Pain Scale, and ICOAP. Arthritis Care Res..

[20] Jensen M.P., Tomé-Pires C., de la Vega R., Galán S., Solé E., Miró J. (2017). What determines whether a pain is rated as mild, moderate, or severe? The importance of pain beliefs and pain interference. Clin. J. Pain.

[21] Breivik H., Borchgrevink P.C., Allen S.M., Rosseland L.A., Romundstad L., Hals E.K., Kvarstein G., Stubhaug A. (2008). Assessment of pain. Br. J. Anaesth..

[22] Shipe M.E., Deppen S.A., Farjah F., Grogan E.L. (2019). Developing Prediction Models for Clinical Use Using Logistic Regression: An Overview. J. Thorac. Dis..

[23] World Bank Bangladesh and South Asia (Developing Only). https://data.worldbank.org/?locations=BD-XN.

[24] Leek J.C., Arif H. (2025). Pregnancy Medications. StatPearls [Internet].

[25] Khattak H.G., Arshad H., Anwar K., Hanif S., Kiani N.N., Sultana B. (2022). Prevalence of musculoskeletal disorders among pregnant women: Cross-sectional study. Pak. J. Med. Health Sci..

[26] Shanshan H., Liying C., Huihong Z., Yanting W., Tiantian L., Tong J., Jiawei Q. (2024). Prevalence of lumbopelvic pain during pregnancy: A systematic review and meta-analysis of cross-sectional studies. Acta Obstet. Gynecol. Scand..

[27] Bakilan F., Zelveci D.D. (2020). Musculoskeletal problems during pregnancy. J. Clin. Med. Kaz..

[28] Zewudie B.T., Temere B.C., Eniyew M.A., Mesfin Y., Tenaw S.G. (2022). Low back pain and associated factors among obstetrics care providers in Ethiopia: A cross-sectional study. BMJ Open.

[29] Onyemaechi N.O., Chigbu C.O., Ugwu E.O., Omoke N.I., Lasebikan O.A., Ozumba B.C. (2021). Prevalence and risk factors associated with musculoskeletal disorders among pregnant women in Enugu, Nigeria. Niger. J. Clin. Pract..

[30] Osazee K., Nnakwe L., Iribhogbe O. (2023). Prevalence of musculoskeletal related morbidity and its perceived impact among pregnant women in a tertiary center. Ibom Med. J..

[31] Gutke A., Boissonnault J., Declercq E., Skaner Y., Östgaard H.C. (2018). Severity and impact of pelvic girdle pain and low back pain in pregnancy: A multinational study. J. Womens Health Phys. Ther..

[32] Anselmo D.S., Love E., Tango D.N., Robinson L. (2017). Musculoskeletal effects of pregnancy on the lower extremity: A literature review. J. Am. Podiatr. Med. Assoc..

[33] Habak P.J., Carlson K., Griggs R.P. (2025). Urinary Tract Infection in Pregnancy. StatPearls [Internet].

[34] Juszczak K., Dybowski B., Holecki M., Hryniewicz W., Klimek H., Kłoda K., Sieroszewski P., Drewa T. (2024). Summary of guidelines on diagnosis, therapy, and management of community-acquired lower urinary tract infections. Cent. Eur. J. Urol..

[35] Sánchez-Polán M., Nagpal T.S., Zhang D., Silva-Jose C., Montejo R., Barakat R. (2023). The influence of physical activity during pregnancy on maternal pain and discomfort: A meta-analysis. J. Pers. Med..

[36] Koukoulithras I., Stamouli A., Kolokotsios S., Plexousakis M., Mavrogiannopoulou C. (2021). The effectiveness of non-pharmaceutical interventions upon pregnancy-related low back pain: A systematic review and meta-analysis. Cureus.

[37] Shiri R., Karppinen J., Leino-Arjas P., Solovieva S., Viikari-Juntura E. (2010). The association between obesity and low back pain: A meta-analysis. Am. J. Epidemiol..

[38] Sharp K.J., Sherar L.B., Kettle V.E., Sanders J.P., Daley A.J. (2022). Effectiveness of interventions to increase device-measured physical activity in pregnant women: Systematic review and meta-analysis of randomised controlled trials. Int. J. Behav. Nutr. Phys. Act..

[39] MacDonald L.A., Johnson C.Y., Lu M.L., Santiago-Colón A., Adam G.P., Kimmel H.J., Napolitano P.G., Saldanha I.J. (2024). Physical job demands in pregnancy and associated musculoskeletal health and employment outcomes: A systematic review. Am. J. Obstet. Gynecol..

[40] Jungari S.B., Paswan B. (2019). What he knows about her and how it affects her? Husband’s knowledge of pregnancy complications and maternal care utilization among tribal populations in India. BMC Pregnancy Childbirth.

[41] Osinuga A., Hicks C., Ibitoye S.E., Schweizer M., Fethke N.B., Baker K.K. (2021). A meta-analysis of the association between physical demands of domestic labor and back pain among women. BMC Women’s Health.

[42] Aldabe D., Lawrenson P., Sullivan J., Hyland G., Bussey M.D., Hammer N., Bryant K., Woodley S.J. (2022). Management of women with pregnancy-related pelvic girdle pain: An international Delphi study. Physiotherapy.

[43] Ifalahma D., Yuliana A., Bakkar Z.A., Wargani R.N., Puspitasari R.A. (2024). Remodeling pregnancy exercises with pelvic rocking exercise as management of back pain in pregnant women. Indones. J. Glob. Health Res..

[44] Akter E., Hossain A.T., Rahman A.E., Ahmed A., Tahsina T., Tanwi T.S., Nusrat N., Nahar Q., El Arifeen S., Chowdhury M.E. (2023). Levels and determinants of quality antenatal care in Bangladesh: Evidence from the Bangladesh Demographic and Health Survey. PLoS ONE.

[45] Ferreira C.W.S., Alburquerque-Sendín F. (2013). Effectiveness of physical therapy for pregnancy-related low back and/or pelvic pain after delivery: A systematic review. Physiother. Theory Pract..

[46] Briggs A.M., Jordan J.E., Kopansky-Giles D., Sharma S., March L., Schneider C.H., Mishrra S., Young J.J., Slater H. (2021). The need for adaptable global guidance in health systems strengthening for musculoskeletal health: A qualitative study of international key informants. Glob. Health Res. Policy.

